# Activity of Anlotinib in the Second-Line Therapy of Metastatic Gastrointestinal Stromal Tumors: A Prospective, Multicenter, In Vitro Study

**DOI:** 10.1093/oncolo/oyac271

**Published:** 2023-02-13

**Authors:** Yongjian Zhou, Chunling Zeng, Xiaofeng Sun, Jun Zhang, Hongyan Qu, Xinhua Zhang, Ye Zhou, Zimin Liu, Xiaojun Wu, Xin Wu, Xuelong Jiao, Lin Shen, Yanbing Zhou, Yuexiang Wang, Jian Li

**Affiliations:** Department of Gastric Surgery, Fujian Medical University Union Hospital, Fujian, People’s Republic of China; CAS Key Laboratory of Tissue Microenvironment and Tumor, Shanghai Institute of Nutrition and Health, University of Chinese Academy of Sciences, Chinese Academy of Sciences, Shanghai, People’s Republic of China; Department of Internal Medicine, Jiangsu Cancer Hospital & Jiangsu Institute of Cancer Research & the Affiliated Cancer Hospital of Nanjing Medical University, Jiangsu, People’s Republic of China; Department of Gastrointestinal Surgery, the First Affiliated Hospital of Chongqing Medical University, Chongqing, People’s Republic of China; The Second Ward of Gastroenterology Department, Harbin Medical University Cancer Hospital, Heilongjiang, People’s Republic of China; Department of Gastrointestinal Surgery, the First Affiliated Hospital, Sun Yat-sen University, Guangdong, People’s Republic of China; Department of Gastric Surgery, Fudan University Shanghai Cancer Center, Shanghai, People’s Republic of China; Department of Oncology, the Affiliated Hospital of Qingdao University, Shandong, People’s Republic of China; Department of Colorectal Surgery, State Key Laboratory of Oncology in South People’s Republic of China, Collaborative Innovation Center for Cancer Medicine, Sun Yat-Sen University Cancer Center, Guangzhou, People’s Republic of China; Department of General Surgery, the General Hospital of the People’s Liberation Army, Beijing, People’s Republic of China; Department of Gastrointestinal Surgery, the Affiliated Hospital of Qingdao University, Shandong, People’s Republic of China; Department of GI Oncology, Laboratory of Carcinogenesis and Translational Research of the Ministry of Education, Peking University School of Oncology, Beijing Cancer Hospital & Institute, Beijing, People’s Republic of China; Department of Gastrointestinal Surgery, the Affiliated Hospital of Qingdao University, Shandong, People’s Republic of China; CAS Key Laboratory of Tissue Microenvironment and Tumor, Shanghai Institute of Nutrition and Health, University of Chinese Academy of Sciences, Chinese Academy of Sciences, Shanghai, People’s Republic of China; Department of GI Oncology, Laboratory of Carcinogenesis and Translational Research of the Ministry of Education, Peking University School of Oncology, Beijing Cancer Hospital & Institute, Beijing, People’s Republic of China

**Keywords:** gastrointestinal stromal tumor, anlotinib, antitumor activity, second-line treatment

## Abstract

**Background:**

Anlotinib is a multi-target tyrosine kinase inhibitor that can effectively inhibit tumor cell proliferation after receptor kinase activation caused by KIT gene mutation.

**Methods:**

We tested the inhibitory effect of anlotinib in GIST cell lines with different gene mutations and evaluated the efficacy of anlotinib for patients with metastatic GIST after imatinib failure in a multicenter, single-arm, phase II study.

**Results:**

In vitro, V654A mutation encoded by KIT exon 13 was intermediately sensitive to anlotinib. Moreover, anlotinib was able to partly suppress the activation loop mutation D820A from exon 17 while another activation loop mutation N822K, also from exon 17, was resistant to anlotinib. From September 2018 to October 2020, 64 patients from 9 Chinese medical centers were enrolled in this study. Seven patients had partial response and 39 patients had stable disease. The median PFS was 8.0 months. There was no statistical significance comparing with PFS of sunitinib second-line therapy at the same period. The most common adverse events related to anlotinib treatment were hypertension, neutropenia, and fatigue.

**Conclusion:**

Anlotinib showed moderate antitumor activity in drug-resistant GIST cell lines in vitro, and good PFS and better tolerance in second-line therapy study.

## Teaser

This article reports on the inhibitory effect of anlotinib in GIST cell lines with different gene mutations, evaluating the efficacy and safety of second-line treatment of GIST with anlotinib compared with second-line treatment with sunitinib in the same period. The clinical trial registration number is NCT04106024.

**Implications for Practice**

This is the first clinical study to evaluate the treatment of GIST with anlotinib. Although no randomized control was conducted, data from patients treated during the same time period were used for comparison, and the results are encouraging. This research data is of positive significance for further research on anlotinib.

## Introduction

Gastrointestinal stromal tumor (GIST) is the most common gastrointestinal stromal tumor.^1^ There were approximately 80%-85% of GISTs-activated mutations in KIT or PDGFRA gene.^2^ Currently, imatinib is the only drug approved for the first-line treatment of metastatic GIST and acquired secondary resistance was ultimately observed in most patients after imatinib resistance.^3-5^ Sunitinib is currently the first choice for the second-line treatment of GIST after the failure of first-line treatment, with a clinical benefit rate of only 50% and a PFS of 6-8 months and several studies showed that GIST with KIT primary or secondary mutations of exon 17 or 18 was not sensitive to sunitinib treatment.^5,6^

Anlotinib is a multi-target tyrosine kinase inhibitor, which can inhibit angiogenesis and tumor growth. It can effectively inhibit KIT, VEGFR, PDGFR, FGFR, and other targets. Meanwhile, it showed certain anti-tumor activity in the treatment of GIST with KIT D816H, V560G, V654A, and PDGFRA D842V mutation.^7,8^ Anlotinib and sunitinib may have different sensitivity to different gene mutation types in the inhibition of GIST. In some studies of different solid tumors, Anlotinib has shown good efficacy and tolerability.^9-11^

In our study, we tested the inhibitory effect of anlotinib in GIST cell lines with different gene mutations, and conducted a prospective multicenter clinical trial to evaluate the efficacy and safety of second-line treatment of GIST with anlotinib, and compared the efficacy with patients receiving second-line treatment with sunitinib in the same period. The clinical trial registration number is NCT04106024.

## Material and Methods

### Reagents and Antibodies

Anlotinib was dissolved with dimethyl sulfoxide (DMSO). Primary antibodies were p-KIT^Y721^ (Cell Signaling Technology (CST), #3391), KIT (Dako, A4502), p-AKT^Ser473^ (CST, #9271), AKT (CST, #9272), p-MAPK^Thr202/Tyr204^ (CST, #9101), MAPK (CST, #9102), and GAPDH (Sigma, G8795).

### Cell Lines

HEK (human embryonic kidney) 293T cells were purchased from the American Type Culture Collection and were maintained in RPMI1640 medium with 10% fetal bovine serum (FBS). https://wx.qq.com/cgi-bin/mmwebwx-bin/webwxgetmsgimg?&MsgID=9173742512830034811&skey=%40crypt_7fcbb234_406a1a7300bb46977b0d1dee5b6f2e7d_GIST430/654 and GIST48 cell lines were generous gifts from professor Jonathan Fletcher at Harvard Medical School and were maintained in IMDM medium containing 10% FBS. The cells were maintained at 37 °C with a 5% CO_2_ humidified atmosphere. All cell lines were verified by Sanger sequencing and whole-exome sequencing assays.

### Mutant KIT Constructs and Transient Transfection

Based on the KIT wild-type (WT) eukaryotic expression vectors, 4 KIT mutants were established with the QuikChange Lightning Site-Directed Mutagenesis KIT (#210518), including V559D + V654A, V559D + D820A, V559D + N822K, A502_Y503dup + N822K. All mutants were confirmed by Sanger sequencing. HEK293T cells were transiently transfected with the above 4 mutants and were treated with anlotinib for 4 h after 40-48 h of normal culture. Protein lysates were prepared for western blotting to test the biochemical activity of anlotinib at inhibiting the KIT kinase.

### Western Blotting

Anlotinib with 5 concentrations, 0, 10, 50, 200, and 500 nmol/L, was added to the 2 GIST cell lines for 4 h when the confluence of the cells were 90%. The whole cell lysates were prepared using a standard protocol. The protein lysate concentrations were measured using a Quick Start Bradford 1 × Dye Reagent (Bio-Rad; #5000205). Electrophoresis and western blotting were performed using standard techniques. The hybridization signals were detected by chemiluminescence (Immobilon Western, Millipore Corporation, MA) and captured using an Amersham Imager 600 imagers (GE Healthcare; #29083461).

### Cell Viability

Viability experiments were carried out with the CellTiter-Glo luminescent assay. Cells were plated at 1-2 × 10^4^ cells per well in a 96-well flat-bottomed plate. Anlotinib with 8 concentrations, 0, 10, 25, 50, 100, 250, 500, and 1000 nmol/L were added to 96-well plates after 48 h. Luminescence was analyzed using a Veritas microplate luminometer.

### Eligibility

Participants were eligible for inclusion if they had recurrent/ metastatic gastrointestinal stromal tumor confirmed by histopathological examination, were 18 years of age or older, have measurable lesions in accordance with RECIST 1.1, and failed the first-line treatment of imatinib or cannot tolerate the adverse reactions, cannot afford the expensive medical expenses of sunitinib and regorafenib or are unwilling to accept their side effects, had an Eastern Cooperative Oncology Group performance status of 0 or 1, have an estimated survival of more than 3 months, and informed consent and signed a written consent. What’s more, the patients have good compliance, and could voluntary follow-up, treatment, laboratory examination, and other research steps as planned.

Exclusion criteria included having resectable localized or metastatic GIST, AST and/ or ALT > 2.5 × ULN, or serum total bilirubin >1.5 × ULN, the absolute count of neutrophil (ANC) was less than 1.5 × 109/L, or platelet <75 × 109/L, or HB <90 g/L, Cr >1.5 × ULN, finding the second primary malignant tumor in the past 5 years, except for skin basal cell carcinoma or cervical carcinoma in situ, finding brain metastasis, spinal cord compression, carcinomatous meningitis, or diseases of brain or pia mater detected by CT or MRI during screening stage, or having any of the following diseases within 12 months before enrollment: myocardial infarction, severe/ unstable angina pectoris, coronary/ peripheral artery bypass grafting, symptomatic congestive heart failure or cerebrovascular accident, being serum HIV antibody positive, participating in other clinical trials at present, being pregnant or nursing, or other reasons may be deemed unsuitable for study participation by the investigator.

### Study Design

This study was a multi-center, single-arm, phase II study to evaluate the efficacy of anlotinib for patients with metastatic GIST after imatinib failure. The primary endpoint was to assess median progression-free survival (PFS) of anlotinib therapy. The secondary endpoints included assessment of overall response rate (ORR), disease control rate (DCR), overall survival (OS), and safety of anlotinib. The DCR was defined as CR + PR + SD, and the RECIST standard is at least 4 weeks. The study also compared the data of this anlotinib trial with second-line sunitinib treatment in patients with metastatic GIST in the real world during the study period.

All procedures performed in this study involving human participants were approved by Beijing Cancer Hospital Ethical Committee. Written informed consent was obtained from the patients before study entry. The study was conducted according to the ethical principles of the Declaration of Helsinki. The clinical trial registration number is NCT04106024.

### Study Drug Administration

Subjects orally took Anlotinib capsules, 12 mg/day, 1 capsule once daily before breakfast by study nurse. It was taken continuously for 2 weeks, followed by 1 week of drug withdrawal, that was, 3 weeks (21 days) as a course of treatment. Continuous administration until disease progression or intolerable adverse reactions.

### Efficacy and Safety

Tumor assessment and response to anlotinib therapy were evaluated according to the Response Evaluation Criteria in Solid Tumors (RECIST v1.1). Tumor responses were assessed by local radiological review. All sites of tumor lesions were investigated using computed tomography at baseline within 3 weeks of enrollment before initiating therapy, then every 6 weeks until disease progression. Physical examination, blood cell counts, and blood biochemistry were carried out at baseline, on day 1 of each cycle. Adverse events were recorded according to Common Terminology Criteria for Adverse Events, version 4.0.

### Detection of KIT and PDGFR Gene Mutations

Tissue samples were provided: fifteen 5-μm thick white slices of GIST tumor tissue.

Peripheral blood sample provision: blood samples, 5 mL anticoagulant and 5 mL non-anticoagulant, will be collected before treatment and at the first efficacy assessment after treatment.

Tissue samples were used for KIT/PDGFRA genetic testing: peripheral blood samples were used to explore predictors of possible association with anlotinib. The genetic detection sites included exons 9, 11, 13, 14, 17, 18 of KIT gene and exons 12 and 18 of PDGFRA.

### Statistical Considerations

Based on the PFS of 6 months in sunitinib phase III study,^6^ we assumed the median PFS of anlotinib treatment could prolong to be 9 months. Assuming a sample with a default rate of 10%, and a one-sided level of significance of 0.05, a 2-sided log-rank test with an overall sample size of 62 subjects achieves 80% power at a 0.05% significance level.

All statistical analyses used the SPSS 19.0 platform. PFS and OS curves were constructed according to the Kaplan–Meier method and were compared using a log-rank test. Frequency and percentage descriptions were used for categorical variables and chi-squared test was conducted to compare the incidence of different events. If the theoretical frequency was lower than 1, Fisher’s exact test was conducted.

## Results

### Potency of Anlotinib for Inhibition of the KIT and KIT Signaling Pathways In Vitro

**Figure 1.**
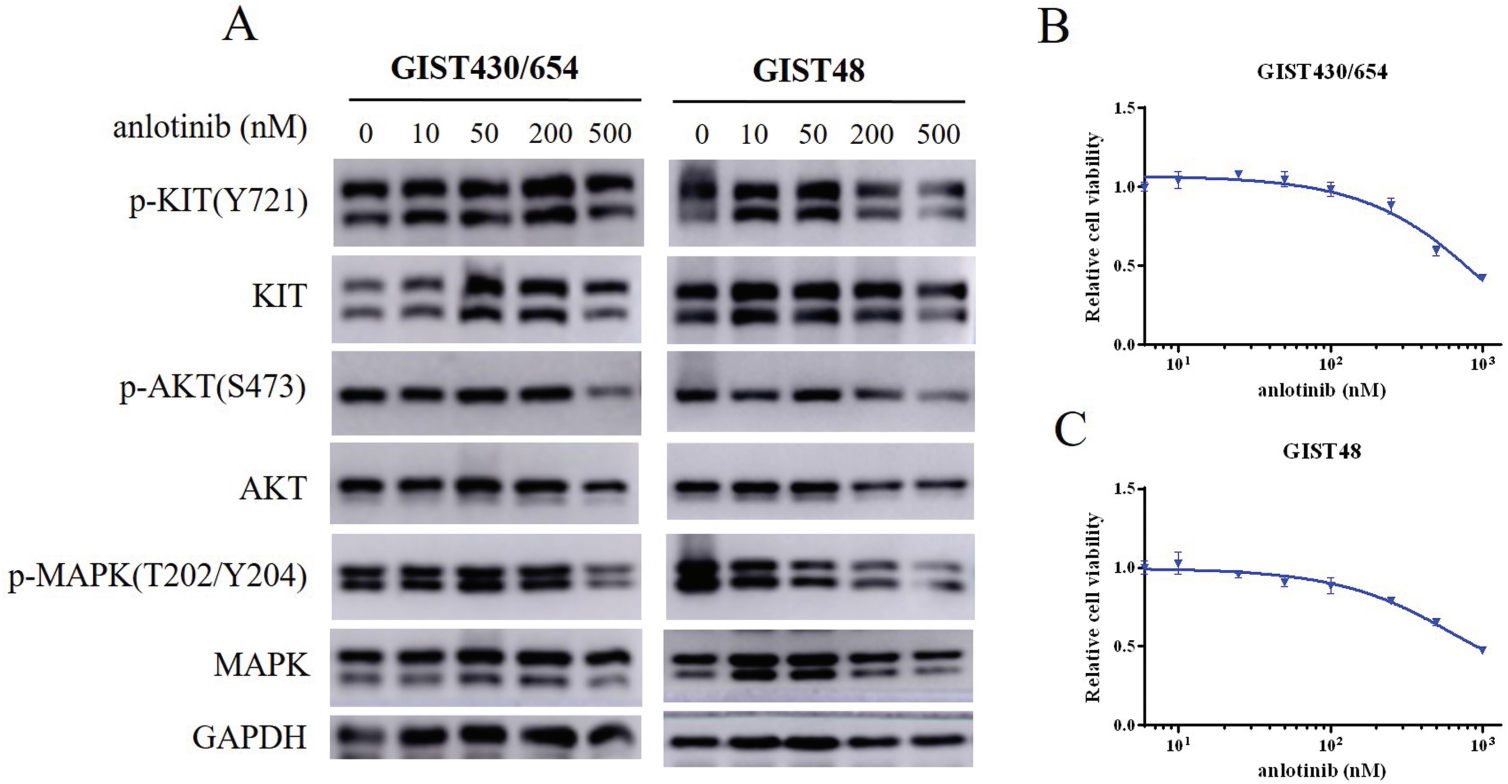
The efficacy of anlotinib against 4 common KIT secondary mutations in HEK293T transient transfection model.

We constructed 4 common secondary mutations to evaluate the efficacy of anlotinib against KIT mutants in HEK293T transient transfection model. As shown in Fig. 1, the most common secondary mutation V654A encoded by exon 13 was intermediately sensitive to anlotinib. Moreover, as shown in Table 2, anlotinib was able to partly suppress the activation loop mutation D820A from exon 17, while another activation loop mutation N822K, also from exon 17, was resistant to anlotinib.

**Table 2.** Drug sensitivity of KIT mutations.

Exons	Mutation	Imatinib	Sunitinib	Regorafenib
Exon 9	AY502-503dup	Sensitive	Sensitive	Sensitive
Exon 11	V560D	Sensitive	Sensitive	Sensitive
Exon 13	V654	Resistance	Sensitive	Resistance
Exon 14	T570	Resistance	Sensitive	Sensitive
Exon 17	D816	Resistance	Resistance	Changeable
Exon 17	D820	Resistance	Resistance	Sensitive
Exon 17	N822	Resistance	Resistance	Sensitive
Exon 17	Y823	Resistance	Resistance	Sensitive
Exon 18	A829P	Resistance	Resistance	Sensitive

**Figure 2.**
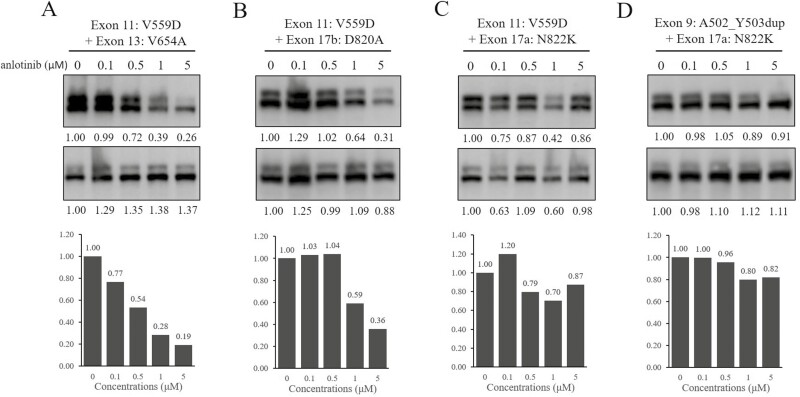
The potency of anlotinib against KIT and KIT signaling pathways in GIST430/654 and GIST48 cell lines.

To provide more evidence about anlotinib efficacy, we assessed the potency of anlotinib against KIT and KIT signaling pathways in GIST cell lines. In accordance with the above results, anlotinib was capable of slightly inhibiting KIT and KIT signaling pathways in GIST430/654 with an IC_50_ of 500 nmol/L. Furthermore, GIST48 was also intermediately sensitive to anlotinib with an IC_50_ of less than 500 nmol/L. However, when we explored the efficacy of anlotinib for decreasing proliferation of imatinib-resistant cell lines including GIST430/654 and GIST48 with a CTG assay, the 2 cell lines were not very sensitive to anlotinib (Fig. 2).

### Patient Characteristics

From September 2018 to October 2020, 64 patients from 9 Chinese medical centers were enrolled in this study. The median age at the time of study entry was 57 years (range, 24-73 years). Forty-six patients (71.9%) were men, and 49 (76.6%) patients had ECOG performance status 1. All the patients had tumor progression after imatinib treatment failure. Among them, 3 patients withdrew from the study before completing one cycle of treatment, 61 patients completed tumor assessment. Fifty-six patients received KIT/PDGFRA genotype examination (Table 1). Fifteen patients underwent secondary gene mutation detection, of which 7 cases detected secondary mutations in exon 17 of KIT gene, including N822K (3 cases), Y823D (2 cases), D820A (1 case) and D816H (1 case), and 2 cases detected secondary exon 13 V654A mutation of KIT gene.

**Table 1.** Clinicopathological characteristics of all the patients receiving anlotinib.

Clinicopathological characteristics	Number (%)
Gender	
Male	46 (71.9)
Female	18 (28.1)
Ages (years)	57 (24-73)
Primary location	
Stomach	20 (31.3)
Small intestinal	35 (54.7)
Other	9 (14.1)
Primary site resection	
Yes	58 (90.6)
No	6 (9.4)
ECOG PS	
0	15 (23.4)
1	49 (76.6)
Primary genotype	
Exon 11 mt	37 (57.8)
Exon 17 mt	3 (4.7)
Exon 9 mt	5 (7.8)
PDGFRA	3 (4.7)
Wild type	11 (17.2)
Unknown	8 (12.5)
PFS of IM first-line therapy	
<12 months	18 (28.1)
>12 months	41 (64.1)
Unknown	5 (7.8)

Remarks: There were some patients with multiple exon mutations, so the total percentage is greater than 100.

In addition, we collected the data of sunitinib second-line therapy during anlotinib trial in study centers as historical retrospective data. Sixty one patients with metastatic GIST received sunitnib second-line therapy from September 2018 to October 2020.

### The Efficacy of Anlotinib

**Figure 3.**
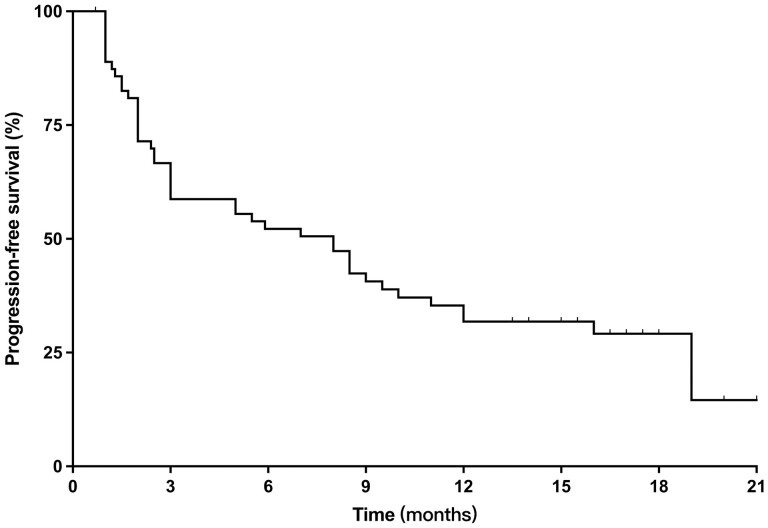
The median PFS of anlotinib second-line therapy in metastatic GIST.

At the end of the study, the clinical trial had met the pre-specified primary endpoint. The median follow-up time was 18.0 months (95% CI, 15.8-21.2). Forty-six patients had GIST progression while 15 patients died and 1 patient was lost to follow-up. Among the 64 patients, 7 (10.9%) patients had partial response, 39 (60.9%) patients had stable disease, 15 (23.4%) patients had disease progression, and 3 patients were not evaluated because of less than one cycle of anlotinib treatment. DCR was 71.8%. The median PFS was 8.0 months (95% CI, 4.7-11.3 months) (Fig. 3) and the median OS was not reached.

### The Efficacy in Different Genotype Subgroups

The median PFS of patients in KIT exon 9 mutation (*n* = 5), KIT exon 11 mutation (*n* = 37), and wild type (*n* = 11) subgroups were 2.0 months (95% CI, 1.2-8.4), 8.0 months (95% CI, 4.6-11.4), and 9.5 months (95% CI, 0.0-24.1), respectively. The median PFS of secondary exon 17 mutation (*n* = 3) treated with anlotinib was 2.5 months (95% CI, 1.217-3.783). The PFS of 2 patients with V654A secondary mutation treated with anlotinib were 3.5 and 5.0 months, respectively. In addition, 1 patient with PDGFRA D842V mutation got 8.5 months PFS and no tumor progression happened and 3 patients with SDHB-deficient GIST had average of 16 months PFS.

### Safety of Anlotinib Therapy

The most common adverse events related to anlotinib treatment were hypertension. Other common adverse events (>10%) include neutropenia, fatigue, anemia, anorexia, hand foot syndrome, nausea, proteinuria, diarrhea, and hemorrhage. Three patients had grade 3 adverse events of hypertension and hand foot syndrome and had temporary termination of treatment. No any patients reduced the dosage of anlotinib therapy because of adverse events. It is worth noting that 7 patients (10.9%) happened grade 1 gastrointestinal bleeding or intratumoral hemorrhage during anlotinib treatment. No treatment-related death happened in this study (Table 3).

**Table 3.** Adverse events of anlotinib treatment related.

Adverse events	All grade (%)	Grades 3-4
Hypertension	26 (40.6)	2 (3.1)
Neutropenia	17 (26.6)	0
Fatigue	14 (21.9)	0
Anemia	13 (20.3)	0
Anorexia	11 (17.2)	0
Hand foot syndrome	11 (17.2)	1 (1.6)
Nausea	9 (14.1)	0
Proteinuria	8 (12.5)	0
Diarrhoea	7 (10.9)	0
Haemorrhage	7 (10.9)	0
Thromobocytopenia	5 (7.8)	0
ALT/AST increased	3 (4.7)	0
Hypothyroidism	3 (4.7)	0
Rash	3 (4.7)	0
Hoarseness	3 (4.7)	0
Bone pain	3 (4.7)	0

## Discussion

**Figure 4.**
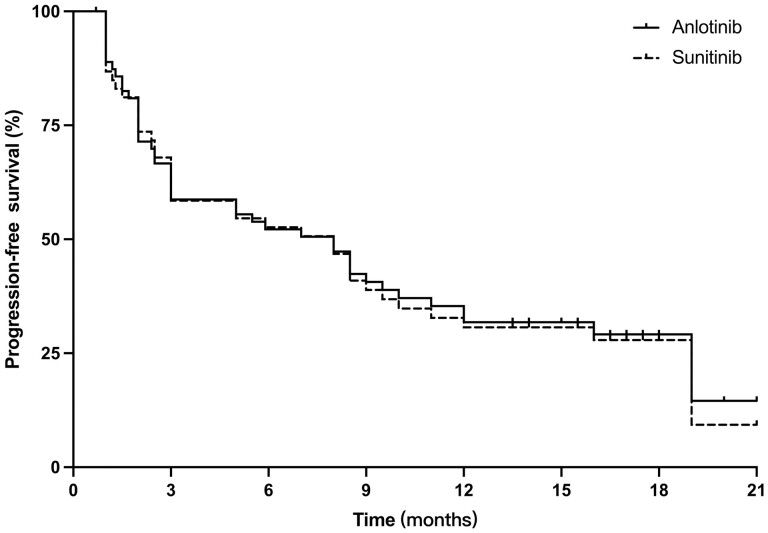
The median PFS of anlotinib comparing with sunitinib in second-line therapy of metastatic GIST.

As a multi-target TKI, anlotinib has shown broader and stronger antitumor activity of in vitro studies than imatinib in KIT mutant GIST cells.^7^ As a reference, we compared to the phase 2 study of sunitinib, the median PFS of sunitinib second-line treatment in 61 patients was 8.0 months (95% CI, 3.7-12.2). There was no statistical significance (*P* = .859) (Fig. 4). Although the superiority in therapeutic efficacy of anlotinib compared to sunitinib did not reached statistical significance as second-line treatment of metastatic GIST, anlotinib still showed good antitumor activity in the present study. Eight months of PFS with anlotinib is an acceptable outcome compared with the outcome of sunitinib phase III clinical trial. Meanwhile, anlotinib treatment showed obvious advantages of safety because only 3 patients had grade 3 adverse events and no patients had dose reduction or interruption due to adverse events. These data suggest that anlotinib is superior to Sunitinib in terms of safety and is unique in terms of therapeutic efficacy.

The result in this study is very similar to the just released phase III study (INTRIGUE) of ripretinib versus sunitinib in GIST second-line treatment.^12,13^ Ripretinib, as an activation loop switch inhibitor, showed significant inhibitory effect on the different KIT mutant GIST cell line and was approved as a fourth-line treatment for metastatic GIST.^14^ Interestingly, PFS of 8.0 months and 8.3 months were obtained in INTRIGUE study of ripretinib and sunitinib, respectively, similar to that of anlotinib in the current study.^12^

There was another interesting phenomenon from subgroup analysis that the effect of anlotinib on GIST with exon 9 mutation was the worst compared with exon 11 mutation, which was just opposite to sunitinib as the second-line treatment for GIST.^5^ In vitro studies, we found that the inhibitory effect of anlotinib in exon 9 mutation combined with exon 17 mutation was poor, which was consistent with the data in this clinical study. In addition, anlotinib showed a good activity on GIST with exon 13 V654A mutation, but unfortunately, we did not detect any patient with V654A mutation in this clinical study to verify. Meanwhile, we found that a patient with KIT gene exon 17 D816H secondary mutation obtained PFS for 9 months, which was consistent with the results of in vitro study.^7^ Anlotinib also showed a good therapeutic effect in the cases of PDGFRA D842V mutation and SDHB- deficient GIST. Ahmad has pointed out that SDH-deficient GISTs show primary resistance to imatinib,^15^ but in our research, the patient with SDHB-deficient GIST (*n* = 3) had an astonishing average of 16 months PFS. It gives us hope for the treatment of patients with SDH-deficiency. Confidence in further research on the anlotinib treatment of patients with specific gene types is increased by these effective cases.

Encouragingly, safety data showed a good tolerance of the toxicities of anlotinib. The incidence of HFS, fatigue, diarrhea, and other adverse events was lower, especially, compared with sunitinib. Another noteworthy phenomenon is that none of the patients reduced the dose of anlotinib due to adverse events. However, a few cases of bleeding events still require further vigilance.

In summary, anlotinib showed moderate antitumor activity in drug-resistant GIST cell lines in vitro, and acceptable PFS and good tolerance were obtained in this prospective study.

## Data Availability

The data underlying this article will be shared on reasonable request to the corresponding author.
